# Outpatient cervical ripening with Foley balloon or mifepristone: Insights into the time taken to reach the active phase of labor

**DOI:** 10.1002/ijgo.70468

**Published:** 2025-08-13

**Authors:** Maria Carvalho‐Afonso, Marília Antunes, Andreia Fonseca, Diogo Ayres‐de‐Campos

**Affiliations:** ^1^ Department of Obstetrics and Gynecology, Medical School University of Lisbon Lisbon Portugal; ^2^ Department of Obstetrics and Gynecology Santa Maria University Hospital Lisbon Portugal; ^3^ CEAUL—Centro de Estatística e Aplicações, Faculdade de Ciências Universidade de Lisboa Lisbon Portugal; ^4^ Department of Obstetrics and Gynecology Hospital Garcia da Orta Almada Portugal

**Keywords:** cervical ripening, Foley catheter balloon, induction of labor, mifepristone, time to active phase of labor

## Abstract

**Objective:**

To estimate the median time elapsed between the start of cervical ripening and the diagnosis of the active phase of labor, according to maternal and pregnancy characteristics, in low‐risk pregnancies submitted to outpatient cervical ripening with a Foley balloon catheter or oral mifepristone.

**Methods:**

This was a secondary analysis of a randomized controlled trial conducted in a European tertiary level hospital, including 101 women with singleton pregnancies, cephalic presentation, and an unfavorable Bishop score. Participants were randomized to receive an intracervical Foley balloon catheter or oral mifepristone (200 mg). The main outcome evaluated was the median time elapsed between the start of the cervical ripening process and the active phase of labor. Cox proportional hazards regression was used to estimate hazard ratios (HR) for factors influencing progression to active labor.

**Results:**

The median interval from the start of cervical ripening to the active phase of labor was 34.3 h (interquartile range [IQR]: 25–50.1). Previous pregnancy (HR: 1.74; 95% CI: 1.18–2.58), gestational age (HR: 1.34; 95% CI: 1.04–1.73), and a permeable cervix at baseline (HR: 2.28; 95% CI: 1.44–3.63) were significantly associated with progression to the active phase of labor. Foley balloon and mifepristone showed comparable median times to the active phase of labor, whereas much shorter durations were observed in those with a permeable cervix at baseline.

**Conclusion:**

Maternal age, gestational age, parity, and permeable cervix at baseline significantly influence the time interval from the start of cervical ripening to the active phase of labor. These may constitute a useful set of information for individualized patient counseling, allowing better alignment of expectations and increased satisfaction with the induction process.

## 1 INTRODUCTION

Women requiring induction of labor (IoL) who have an unfavorable Bishop's score on vaginal examination, are known to benefit from “cervical ripening”, a process that promotes cervical softening, effacement, and dilation.^1^ This process has traditionally been performed in hospital settings, but outpatient use has recently emerged as a viable and safe alternative, offering as potential benefits improved patient satisfaction, cost saving, and less use of hospital resources.^2^, ^3^, ^4^, ^5^

Mechanical methods stand out as the most widely used methods of outpatient cervical ripening, because they have similar efficacies and better maternal and fetal safety profiles than prostaglandins.^6^, ^7^, ^8^ Mifepristone has recently emerged in the scientific literature and in localized clinical practices as a useful cervical ripening agent, due to its antiprogesterone effects, its ability to enhance myometrial sensitivity to uterotonics, and its capacity to induce mild uterine contractility.^9^, ^10^

One of the most challenging aspects of cervical ripening for pregnant women is the uncertainty regarding the time taken until the active phase of labor is reached. This interval has been reported to be widely variable.^11^, ^12^

The aim of the study was to estimate the median time elapsed between the start of cervical ripening and the diagnosis of active phase of labor, according to several maternal and pregnancy characteristics. All women had low‐risk pregnancies and were undergoing outpatient cervical ripening with a Foley balloon catheter or oral mifepristone.

## 2 MATERIALS AND METHODS

This was a secondary analysis of an open‐label, randomized controlled trial (RCT) conducted in a European tertiary care university hospital.^13^ All participants in the parent trial were undergoing outpatient cervical ripening, and were randomized to receive mifepristone 200 mg per os, or an intracervical Foley catheter balloon 16 Fr/Ch 5.3 mm (Covidien, Dublin, Ireland). The inclusion criteria were: singleton pregnancies, gestational age between 37 and 42 weeks, cephalic presentation, and a Bishop score <6 at baseline. Women with contraindications to vaginal birth, prior uterine scars, prelabor rupture of membranes, hypertensive or metabolic disorders, or other high‐risk maternal or fetal conditions were excluded. The trial received approval from the Hospital Research Ethics Committee (04/2019) and was registered on ClinicalTrials.gov (NCT04271722). In the parent trial, written informed consent was obtained from all women who expressed interest in participating. This secondary analysis was conducted in compliance with ethical standards, using anonymized data to protect participant confidentiality.

All women were asked to return to the hospital for re‐evaluation on the morning following the cervical ripening procedure, that is, 18–24 h after its start. They were also instructed to call the labor ward if they experienced regular uterine contractions, rupture of the membranes, vaginal bleeding, or reduced fetal movements. If Bishop's score on re‐evaluation was ≥6, intravenous oxytocin and amniotomy were proposed. If Bishop's score on re‐evaluation was <6, in‐hospital prostaglandins were proposed for a maximum of two cycles. Failed IoL with oxytocin was diagnosed when regular contractions were documented, but no relevant cervical changes occurred after 24 h without amniotomy, or 12 h with amniotomy.^14^ Failed IoL with prostaglandins was diagnosed when Bishop's score remained <6 after two cycles.

The median times elapsed between the start of cervical ripening and the diagnosis of active phase of labor were evaluated, defining the latter as the appearance of regular uterine contractions, together with a cervical dilatation of ≥4 cm, and ≥ 80% effacement.

Kaplan–Meier method was used to estimate the survival curves and log rank tests were used to compare the median times between cervical ripening and the active phase of labor, censoring women who required a cesarean delivery before the latter occurred. Cox proportional hazards regression model was used to estimate the hazard ratio (HR) for the time taken to reach the active phase of labor. Several stratified analyses examined the effect of specific risk factors on this parameter (type of treatment, age, race, body mass index [BMI, calculated as weight in kilograms divided by the square of height in meters] weight gain, parity, gestational age and permeable cervix at baseline) using a stepwise top‐down approach. To confirm that there were no violations that could bias the effect estimate in Cox regression analysis, the proportional hazard for each risk factor was tested. As gestational age violated this assumption, there was a need for stratification in the final model. Using the best fit model derived from Cox proportional hazard regression, the median time between cervical ripening and the active phase of labor was estimated according to different maternal ages (25, 30, 35 and 40), parity (nulliparous and parous), gestational age (37^+0^–39^+6^ weeks and ≥40 weeks) and permeable cervix (yes/no) at baseline. All analyses were performed using R Statistical Software, version 4.4.0, and RStudio version 2024.04.1.

## 3 RESULTS

A total of 101 women were enrolled and randomized in the trial, 48 allocated to the mifepristone group, and 53 to the Foley balloon catheter group.

The main characteristics of the study population are displayed in Table 1. The majority of women were white and nulliparous, with a normal BMI. By far the most common reason for IoL was a gestational age ≥41 weeks.

**TABLE 1:** Main demographic and obstetric characteristics of the study population, which incorporated both allocation groups of a previously published randomized controlled trial.^13^

Characteristics	*N* = 101
Maternal age, years, median (IQR)	31 (28–36)
Race, *n* (%)	
White	87 (86.1)
Black	13 (12.9)
Asian	1 (1.0)
Pre‐gestational BMI, median (IQR)	23.9 (21.6–27.1)
Weight gain in pregnancy (kg), median (IQR)	14 (11–18)
Nulliparous, *n* (%)	74 (73.3)
Gestational age at IoL (weeks), median (IQR)	40 (40–41)
Indication for IoL, *n* (%)	
Post term pregnancy	84 (83.2)
Maternal age ≥40 years	9 (8.9)
Suspected fetal macrosomia	6 (5.9)
Other	2 (2.0)
Bishop score at baseline, median (IQR)	3 (2–4)
Permeable cervix at baseline, *n* (%)	48 (47.5)

*Note*: BMI, calculated as weight in kilograms divided by the square of height in meters.

Abbreviations: BMI, body mass index; IQR, interquartile range; IoL, induction of labor.

Table 2 displays the main outcomes of the cervical ripening process, including labor and delivery. The median time from the start of cervical ripening to the diagnosis of active phase of labor was 34.3 h, while the median time to delivery was 44.0 h. The majority of women (58.42%), delivered within 48 h. In four cases (3.96%), a cesarean section was performed due to failed IoL.

**TABLE 2:** Delivery outcomes and labor characteristics of the study population, which incorporated both allocation groups of a previously published randomized controlled trial.^13^

Outcomes	*N* = 101
Onset of labor within 24 h or BS ≥6 at first re‐evaluation, *n* (%)	32 (31.7%)
BS at first re‐evaluation, median (IQR)	5 (3–6)
Change in BS, median (IQR)	2 (0–3)
Interval from cervical ripening to active labor, h, median (IQR)	34.3 (25–50.1)
Interval from cervical ripening to delivery, h, median (IQR)	44.0 (32–60.8)
Use of oxytocin augmentation, *n* (%)	44 (43.6%)
Delivery within 24 h after cervical ripening, *n* (%)	14 (13.9%)
Delivery within 48 h after cervical ripening, *n* (%)	59 (58.4%)
Failed induction of labor, *n* (%)	4 (4.0%)
Mode of delivery, *n* (%)	
Vaginal delivery	65 (64.4%)
Instrumental vaginal delivery	32 (31.7%)
Cesarean delivery	36 (35.6%)
Cesarean delivery before active stage, *n* (%)	12 (11.9%)

Abbreviations: BS, Bishop score; IQR, interquartile range.

The determinants affecting the time elapsed between the start of cervical ripening and the active phase of labor, using Cox proportional hazards regression analysis, are listed in Table 3. Maternal age was associated with a HR of 0.95, meaning that for each additional year of the mother's age, the hazard of reaching the active phase of labor decreases by 5%. Conversely, higher gestational age, previous delivery, and permeable cervix at baseline were associated with increased hazards of reaching the active phase of labor (74%, 34% and 128%, respectively).

**TABLE 3:** Factors affecting the time interval from cervical ripening to the active phase of labor (Cox regression analysis).

	HR	95% CI	*P* value
Maternal age (years)	0.95	0.92–0.98	0.002
Previous delivery	1.74	1.18–2.58	0.005
Gestational age (weeks)	1.34	1.04–1.73	0.023
Permeable cervix at baseline	2.28	1.44–3.63	0.0004

Abbreviations: CI, confidence interval; HR, hazard ratio.

Using the best fit model, the median times from the start of cervical ripening to the active phase of labor were estimated, in accordance with these four factors, for cervical ripening with the Foley balloon catheter and mifepristone (Table 4).

**TABLE 4:** Estimated median time (h:min) from the start of cervical ripening to the active phase labor according to the method (Foley balloon catheter or mifepristone), parity, maternal age, gestational age and cervical status.

	Permeable cervix	Maternal age			
		25–29 years	30–34 years	35–39 years	40 years
*Foley balloon catheter*					
Nulliparous					
37–40 weeks	No	56:00	56:40	57:00	60:15
	Yes	37:37	44:00	54:04	56:00
≥40 weeks	No	34:20	40:00	50:00	59:30
	Yes	26:00	29:30	32:00	34:00
Multiparous					
37–40 weeks	No	37:54	44:00	54:04	56:40
	Yes	34:45	37:00	37:54	37:54
≥40 weeks	No	29:30	32:00	34:00	39:15
	Yes	18:00	23:00	26:00	29:00
*Mifepristone*					
Nulliparous					
37–40 weeks	No	56:40	57:00	77:36	77:40
	Yes	44:00	54:04	56:00	56:40
≥40 weeks	No	40:00	50:00	59:30	64:20
	Yes	31:00	32:00	34:20	40:00
Multiparous					
37–40 weeks	No	44:00	56:00	56:40	57:00
	Yes	37:00	37:54	37:54	44:00
≥40 weeks	No	32:00	34:00	40:00	49:00
	Yes	24:00	26:00	29:30	32:00

## 4 DISCUSSION

### 4.1 Main findings

Higher maternal age was associated with longer time intervals between the start of cervical ripening and the diagnosis of active phase of labor. Conversely, higher gestational age, higher parity, and permeable cervix at baseline were associated with lower times needed to reach the active phase of labor.

Permeable cervix at baseline had the highest positive effect (HR: 2.28; *P* = 0.0004). In nulliparous women at 37–40 weeks of gestation, outpatient cervical ripening methods required circa 56 h before reaching the active phase of labor, but for those with a permeable cervix, this time was reduced to 37–44 h.

### 4.2 Strengths and limitations

To the best of our knowledge, this is the first study to estimate the median times taken from the start of outpatient cervical ripening to the diagnosis of active phase of labor, according to maternal and pregnancy characteristics. The data used for these estimations were derived from a single center randomized clinical trial, ensuring the use of a uniform clinical protocol. Data extraction from an obstetric‐specific electronic clinical record system allowed a rigorous evaluation of all clinically measured parameters.

On the other hand, the relatively small sample size limits the robustness of the results. In addition, the single center nature of the study, while beneficial for protocol consistency, may not adequately represent clinical practices in other settings, and limits the generalizability of the results to other patient populations.

### 4.3 Interpretation

Direct comparison of our results with previously published studies was constrained by methodological differences, particularly in the cervical ripening methods employed, the definitions of time‐related outcomes, and the organization of induction protocols and clinical care pathways. Nonetheless, the CHOICE study—a large multicenter observational study conducted across 26 maternity units in the UK—offers a valuable point of reference.^15^ This study compared outpatient Foley balloon catheter (*n* = 515) with inpatient vaginal dinoprostone (*n* = 4332), reporting a median time from induction onset to labor ward admission of 31.3 h (interquartile range [IQR]: 22.3–52.0) for the Foley group and 24.8 h (IQR: 12.4–48.6) for the dinoprostone group. Among nulliparous women, median times were 32.7 h (IQR: 22.3–52.2) for Foley and 27.0 h (IQR 13.8–51.0) for dinoprostone. Notably, time to labor ward admission with Foley did not vary significantly by parity but showed a clear reduction with increasing gestational age. This pattern was even more pronounced with dinoprostone, likely reflecting the dual effect of prostaglandins in promoting both cervical ripening and uterine contractility.

Based on our cohort data, the estimated median time from the start of cervical ripening to the active phase of labor among nulliparous women who received the Foley balloon catheter would range from 37.6 to 60.3 h, depending on maternal age, gestational age, and cervical status. Unlike the CHOICE study, which used labor ward admission as a proxy for the onset of active labor, our study clinically defined and directly assessed the active phase, enabling greater accuracy and reducing the risk of misclassification.

The benefits of combining mechanical and pharmacological methods for cervical ripening—particularly in reducing the time to active labor and delivery—have been recent demonstrated. A Finnish multicenter randomized controlled trial involving 273 nulliparous with late and post‐term pregnancies showed that a combination of Foley balloon catheter and oral misoprostol significantly shortened the time from induction to both active labor (median 10.4 h; IQR: 6–23.6) and delivery (median 21.7 h; IQR: 15.1–33.2), compared to either method used alone. However, this combined approach required inpatient admission, limiting its applicability in outpatient settings.^16^

Pregnancy characteristics associated with longer or shorter durations of the cervical ripening process may constitute a useful set of information for individualized patient counseling, allowing better alignment of expectations and increased satisfaction with the labor induction process. A large retrospective cohort study involving 95 051 women who had recently delivered, reported that IoL is associated with a higher risk of poor childbirth experiences, and there is ample evidence that the latter can have a significant impact on future reproductive decisions.^17^, ^18^, ^19^

Effective counseling is essential to address pregnant women's uncertainties surrounding the cervical ripening process. A recent postnatal survey in the UK reported that only 50% of women undergoing IoL fully understood what to expect during the process.^20^ Similarly, a recent study from Finland identified prolonged duration of IoL as the second most important factor for reporting a poor childbirth experience, surpassed only by pain.^21^ Providing clear, individualized information about expected timelines may help to align expectations and thus have a positive impact on the emotional well‐being of women.

Importantly, a recent individual participant data meta‐analysis including 2593 women reinforced that outpatient cervical ripening with mechanical methods is as safe and effective as inpatient strategies.^22^ These data are aligned with growing policy efforts to support women's preferences for less medicalized birth experiences.

The slightly longer time intervals needed to reach the active phase of labor when using mifepristone may reflect its unique pharmacological mechanisms. The Foley balloon catheter induces cervical ripening by physical pressure leading to cervical dilatation and by separating the decidua from the amnion, causing local prostaglandin production, enhancing stromal breakdown, and increasing the myometrium fibers responsiveness to oxytocin and prostaglandins. On the other hand, mifepristone primarily blocks progesterone action by binding to its intracellular receptor, thus eventually causing softening and dilation of the cervix and a mild increase in uterine activity.^9^, ^10^

Despite these differences, mifepristone remains an interesting option for outpatient cervical ripening because of its ease of administration and potential to increase patient comfort and satisfaction. Further research is needed to explore whether repeated doses or tailoring of the dose according to maternal characteristics can optimize efficacy.

## 5 CONCLUSIONS

Maternal age, gestational age, parity, and permeable cervix at baseline significantly influence the time from the start of cervical ripening to the active phase of labor. These may constitute a useful set of information for individualized patient counseling, allowing better alignment of expectations and increased satisfaction with the induction process. Future research is needed to validate these findings in other hospital settings and in different populations.

## AUTHOR CONTRIBUTIONS

MCA and MA conceived the original idea for the study. MCA and DAC wrote the study manuscript. MA led the statistical analysis, with support from MCA. All authors reviewed the text extensively and agreed on its final version.

## FUNDING INFORMATION

The work by MA was partially funded by FCT – Fundação para a Ciência e a Tecnologia under the project UIDB/00006/2025.

## CONFLICT OF INTEREST STATEMENT

The authors have no conflicts of interest to declare.

## Data Availability

Research data are not shared.
